# Porous polymer bilayer with near-ideal solar reflectance and longwave infrared emittance

**DOI:** 10.1515/nanoph-2023-0707

**Published:** 2024-01-17

**Authors:** Yung Chak Anson Tsang, Nithin Jo Varghese, Mathis Degeorges, Jyotirmoy Mandal

**Affiliations:** Department of Civil & Environmental Engineering, Princeton University, Princeton, NJ, USA; Institut National des Sciences Appliquées de Lyon, Lyon, France; Princeton Materials Institute, Princeton University, Princeton, NJ, USA

**Keywords:** metamaterials, radiative cooling, sustainability, porous polymers, superwhite, nanophotonic design

## Abstract

This study explores the optical design of a daytime radiative cooler with near-ideal solar reflectance and longwave infrared (LWIR) emittance through materials selection and nanostructuring. Focusing on polymers as a materials platform, we introduce a bilayer architecture, comprising a porous poly(vinylidene fluoride-co-hexafluoropropene) (P(VdF-HFP)) topcoat that serves as a low-index LWIR emissive effective medium, over a nanofibrous, solar scattering polytetrafluoroethene underlayer. This novel configuration yields a superwhite coating with a near-ideal solar reflectance of >0.99, and a blackbody-like near-normal and hemispherical LWIR emittances of ∼0.98 and ∼0.96 respectively. Under humid and partially cloudy sky conditions unfavorable for radiative heat loss, these values enable the bilayer radiative cooler to achieve a sub-ambient of 2.3 °C. Given that the porous polymer bilayer uses scalable fabrication processes and commercially available materials, it holds significant promise for device-scale, as well as building thermoregulation applications.

## 1 Introduction

Radiative cooling of objects under the sky involves a spontaneous and net radiative heat emission from earth to space in the wavelengths where the atmosphere is transparent – primarily through longwave infrared (LWIR, *λ* ∼ 8–13 μm) transmission window of the atmosphere. In the daytime, it also involves a reflection of solar wavelengths (*λ* ∼ 0.3–2.5 μm). A sky-facing terrestrial surface that radiates LWIR heat and reflects sunlight sufficiently well can spontaneously lose between ∼10 and 150 Wm^−2^ heat to space, and cool to significantly sub-ambient temperatures. This ‘zero-energy, zero-carbon’ functionality is increasingly regarded as a sustainable way to cool terrestrial environments and objects, especially as the effects of climate change manifest around us.

Due to its promise, radiative cooling have been extensively explored in recent decades, yielding a variety of materials, including polymers, dielectrics, metals and various combinations and architectures of those materials, such as porous films, composites, and photonic stacks [1]–[8]. By contrast, the limits of optical performance of these materials and approaches – namely the solar reflectance *R*_solar_ and LWIR emittance *ɛ*_LWIR_, have been less explored. However, this is an important topic of study, both from a fundamental optical design perspective, and for achieving optimal cooling performance.

In this work we consider a subset of radiative cooling materials – polymers – and explore how the optical performance of polymeric radiative coolers can be pushed to near-ideal levels. Our optical and material design considerations lead us to a bilayer architecture, consisting of porous poly(vinylidene fluoride-co-hexafluoropropene) (P(VdF-HFP)) topcoat which acts as a low-index, rough LWIR effective medium and a modest solar scatterer, and a nanofibrous polytetrafluoroethene (PTFE) underlayer which is a highly efficient solar scattering medium. The differing behavior of our design’s microstructure in the solar and thermal infrared (TIR, *λ* ∼ 4–20 μm) yields an *R*_solar_ ∼ 0.991, a near-normal *ɛ*_LWIR,⊥_ ∼ 0.98, and hemispherical *ɛ*_LWIR_ ∼ 0.96. These near-ideal values enable the design to attain sub-ambient cooling of 2.3 °C even under humid, partially cloudy skies. Since the design can be conveniently made using established materials and fabrication techniques, we believe that it can be used for device applications like cooling panels.

## 2 Achieving near-ideal *R*_solar_ and *ɛ*_LWIR_: optical considerations

An ideal sky-facing radiative cooler, whether it is a broadband or selective LWIR emitter, has an *R*_solar_ = 1 and *ɛ*_LWIR_ = 1 [9]. Creating a design that approaches both of these limits is difficult. A brief survey of the literature indicates that while there is an abundance of radiative coolers with *R*_solar_ and *ɛ*_LWIR,⊥_ > 0.90, designs with *R*_solar_ and *ɛ*_LWIR,⊥_ > 0.95 are few, and those approaching *R*_solar_ and *ɛ*_LWIR,⊥_ = 1 are rarer still. Near those limits, fundamental material and structural properties limit what designs limitations can achieve. For instance, radiative cooler architectures that have a solar-transparent LWIR emitter on a solar reflective metal like silver, rarely have *R*_solar_ > 0.96 [2], [3], because of the intrinsic *R*_solar_ of silver (∼0.97) and the fact that a less than perfectly smooth silver surface and the intrinsic absorption of the emitter above lower the reflectance from the intrinsic value. Consequently, most radiative cooling designs with *R*_solar_ > 0.96 are thick, optically inhomogeneous porous polymers and composites that scatter and reflect light [1], [7], [10], [11], although intrinsic absorption of materials limit their *R*_solar_ as well.

Similar material limitations also occur for *ɛ*_LWIR_. A survey of the literature indicates that emitters with *ɛ*_LWIR,⊥_ > 0.90 are quite common, but emitters with *ɛ*_LWIR,⊥_ > 0.95 are few [12]. If we consider the true, hemispherical emittance *ɛ*_LWIR_, which is typically lower than *ɛ*_LWIR,⊥_ but is seldom reported, designs with *ɛ*_LWIR_ > 0.95 are exceptionally rare [12]. One reason behind this is the often-overlooked LWIR surface reflectance of radiative coolers. Although most non-metallic materials used in radiative cooling designs are good intrinsic emitters, their surface reflectance, which is complementary to their emittance, limits *ɛ*_LWIR_. Many radiative cooling designs have planar or microscale-smooth surfaces [2], ], which have a significant surface reflectance, particularly at high angles, because of the refractive index contrast at the air-emitter interface. By contrast, designs with microstructured surfaces have lower surface reflectance and higher *ɛ*_LWIR_ [1], ]. Another factor that applies to both smooth and rough surfaces, is the emitter’s refractive index. The use of strong LWIR emissive materials like SiO_2_ in radiative cooling designs also means that their refractive index is higher than that of air (*n*–1), as dictated by the Kramers–Kronig relations. This can lead to a high backscattering of light from heterogeneous media or high reflections off smooth surfaces, again limiting *ɛ*_LWIR_.

**Figure 1:**
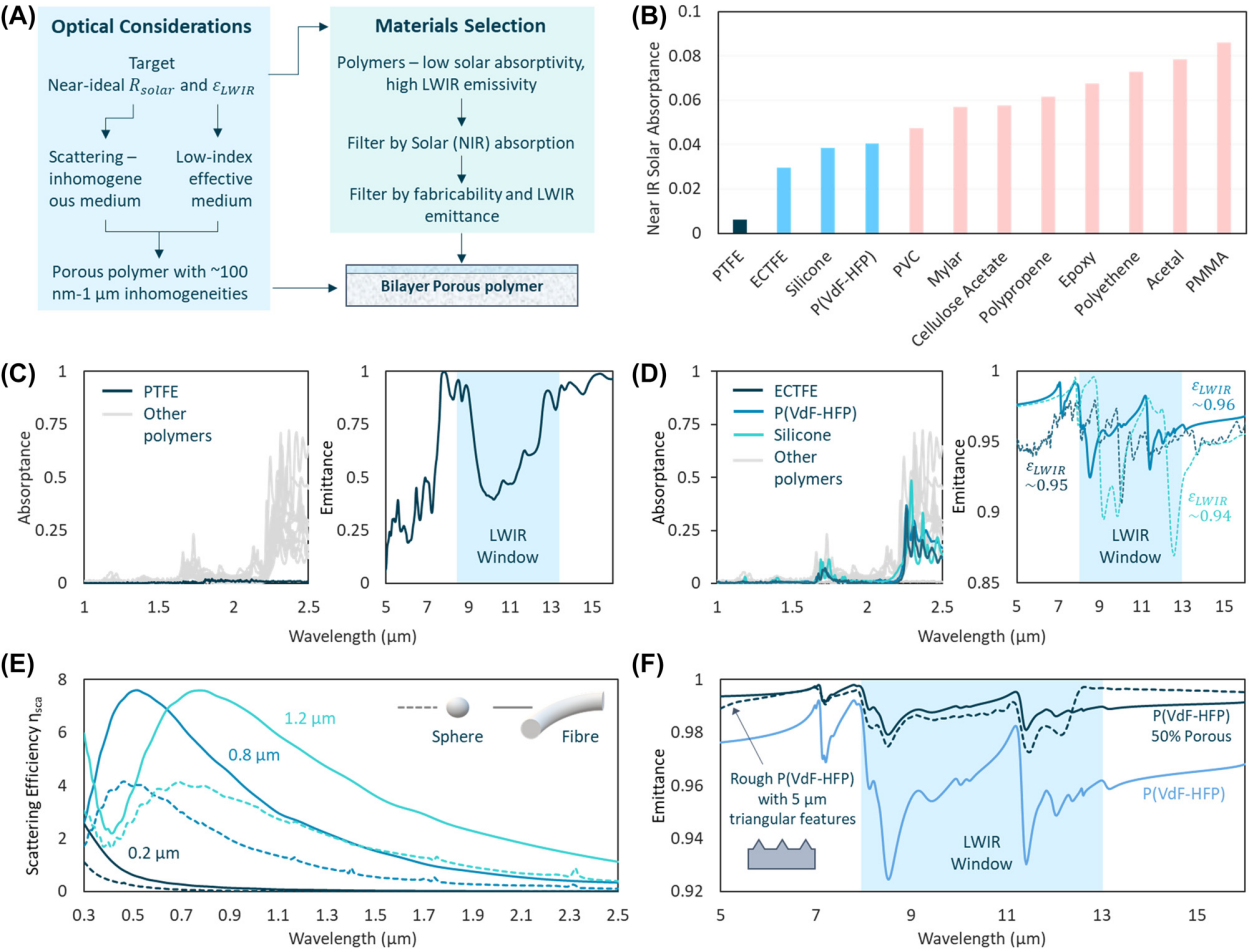
Materials design process for ePTFE-P(VdF-HFP) bilayer structure. (A) Flowchart of optical and materials considerations for the bilayer porous polymer structure. (B) Bar chart highlighting the NIR-to-SWIR absorptance of 12 commercially available polymers. (C) (Left) NIR-to-SWIR absorptance and (Right) TIR emittance spectrum of PTFE. (D) (Left) NIR-to-SWIR absorptance and (Right) TIR emittance of ECTFE, P(VdF-HFP) and silicone. (E) Scattering efficiency of PTFE nanoparticles and fibers of different diameters. (F) TIR emittance of P(VdF-HFP) in bulk, rough and porous forms.

We sought to design a radiative cooler which circumvents these issues through careful choice of materials and microstructure (Figure 1A). Since the use of metal mirrors limit *R*_solar_, we first opted to use an optically heterogeneous medium with ∼0.5–1 μm features to scatter sunlight. Given that features in that size range are Mie scatterers in the solar wavelengths [1], [18], in the absence of absorption, they could theoretically yield *R*_solar_ = 1 if the scattering medium is sufficiently thick.

Promisingly, since the ∼0.5–1 μm heterogeneities are roughly an order of magnitude or smaller than the LWIR wavelengths, the heterogeneous medium could effectively act as a homogenous medium in the LWIR wavelengths. Thus, as long as the scattering and non-absorption in the solar wavelengths were kept intact, we could choose materials and morphologies to create an effective medium with a high *ɛ*_LWIR_. Crucially, this would enable us to go beyond the limitations of single materials that are intrinsically solar transparent but LWIR emissive. If one constituent of the heterogenous medium was a low-index material, this would not only help scatter sunlight, but also lower the effective LWIR refractive index of the medium towards that of air, taking *ɛ*_LWIR_ close to 1. In fact, if the other constituent(s) of the medium were higher index, solar-transparent, LWIR-emitters, this constituent could simply be air. In other words, a nano-to-microporous media with ∼0.5–1 μm features, and containing air in the pores, could hypothetically achieve near-ideal *R*_solar_ and *ɛ*_LWIR_.

## 3 Achieving near-ideal *R*_solar_ and *ɛ*_LWIR_: materials selection

With the general design in mind, we searched for materials that have a near-zero solar absorptivity, and high LWIR emissivity. Polymers, which are generally transparent to sunlight, radiate TIR light due to their molecular vibrations, and are highly processable into porous forms, are promising in this regard, and have long been used as thermal emitters [1], [2], [8], [11], ]. For this study, we investigated a wide range of commercially available polymers, and systematically screened them by their near-to-shortwave infrared (NIR-to-SWIR, *λ* ∼ 1–2.5 μm) solar absorptance, and then by their *ɛ*_LWIR_. The decision to first screen by NIR-to-SWIR absorptance was because of the large magnitude of peak solar intensity (>1000 Wm^−2^) relative to the LWIR cooling potential (10–150 Wm^−2^), and that polymers primarily absorb in the NIR-to-SWIR [7].

Figure 1B shows the twelve least solar absorptive polymers among the polymers we studied. To our knowledge, this is the first systematic comparison of their radiative cooling performance. As evident, polytetrafluoroethene (PTFE, (C_2_F_4_)_*n*_), whose C–C and C–F bonds have minimal absorption in the solar wavelengths, has the lowest solar absorptance among all polymers (Figure 1C). Ethylene-chlorotrifluoroethylene (ECTFE), silicone and P(VdF-HFP) have the second, third and fourth lowest absorptances respectively, with the rest of the polymers having increasingly higher absorptances (Figure 1D). An examination of the chemical structures of these polymers reveals that near-IR absorptances increases with the relative abundance of CH, CH_2_ and CH_3_ groups in the molecular structure, particularly evident in the case of polyethene and polypropene, and additionally, C=O and C–OH groups, in the case of acetal and PMMA. Other bonds, in particular, C–C and C–F bonds, have little NIR-to-SWIR absorption, which is why polymers like PTFE and P(VdF-HFP) absorb little sunlight (Figure 1C and D).

PTFE’s exceptionally low absorptance could make it a near-ideal solar reflector when made porous, as optical scattering in the absence of absorption can lead to a high reflectance (Figure 1E). However, its molecular composition lacks strong vibrational modes precisely in the LWIR wavelengths where they are needed (Figure 1C). Thus, for weakly absorptive porous PTFE, scattering-induced reflectance would lead to a low *ɛ*_LWIR_. ECTFE, Silicone and P(VdF-HFP), which are highly emissive (Figure 1D), could be potential alternatives. However, the intrinsic NIR-to-SWIR absorptance of these materials, even when lower than most polymers, could limit *R*_solar_ of porous films made from them. This has been observed for porous P(VdF-HFP) and silicone, whose *R*_solar_ plateau at ∼0.98 for thick films [1], [25].

These considerations led us to a bilayer architecture, comprising of a solar reflective porous PTFE underlayer, and an LWIR emissive porous polymer topcoat (Figure 1A), which overcomes the above limitations. Central to our design is the fact that absorptances of polymers are typically much stronger in the LWIR than NIR-to-SWIR. This would enable a thin topcoat of a porous LWIR-emissive polymer to enhance *ɛ*_LWIR_ far beyond that of PTFE, while minimizing solar absorptance. The solar reflective PTFE underlayer would, at the same time, maximize *R*_solar_ within the bounds of the solar absorption of the topcoat. Since ECTFE, Silicone and P(VdF-HFP) all have high emittances and similarly NIR-to-SWIR absorptance, we considered them for emissive layer. A comparison of the emissivities of the polymers shows that P(VdF-HFP) has the highest emissivity in the LWIR (Figure 1D). Furthermore, in a previous work [1], we demonstrated that P(VdF-HFP) could be conveniently made into a porous form with near-ideal *ɛ*_LWIR_. These led us to choose a thin, porous P(VdF-HFP) film as the topcoat.

Figure 1E and F shows the functionality of our optical design, simulated using Lumerical FDTD software (SI, Section 8). Nano-to-microporous polymers usually have fibrous or bicontinuous/closed air void morphologies. Scattering efficiencies of PTFE filaments and spherical voids in PTFE matrix (*n*∼1.38) show that an array of fibers or voids with nano-to-microscale (∼0.2–1.2 μm) sizes scatter all solar wavelengths (Figure 1E). In the absence of intrinsic absorption (Figure 1C), this would yield a high *R*_solar_. The same nano-to-microscale features can lead to microscale surface roughness, and provided that they are ≲10× smaller than the LWIR wavelengths, cause the porous polymer to behave as a low-index effective medium. Simulation of bulk P(VdF-HFP) with surface roughness of ∼5 μm shows that surface roughness has an antireflective effect, leading to a higher emittance than the emissivity of smooth bulk P(VdF-HFP) (Figure 1F). To simulate the effective medium behavior, we calculated the effective complex refractive index of 50 % porous P(VdF-HFP) using Maxwell-Garnett effective medium theory, and calculated its emissivity (SI, Section 4). As shown in Figure 1F, this also leads to a high emittance, as would be expected of a low-index material.

## 4 Radiative cooler fabrication

**Figure 2:**
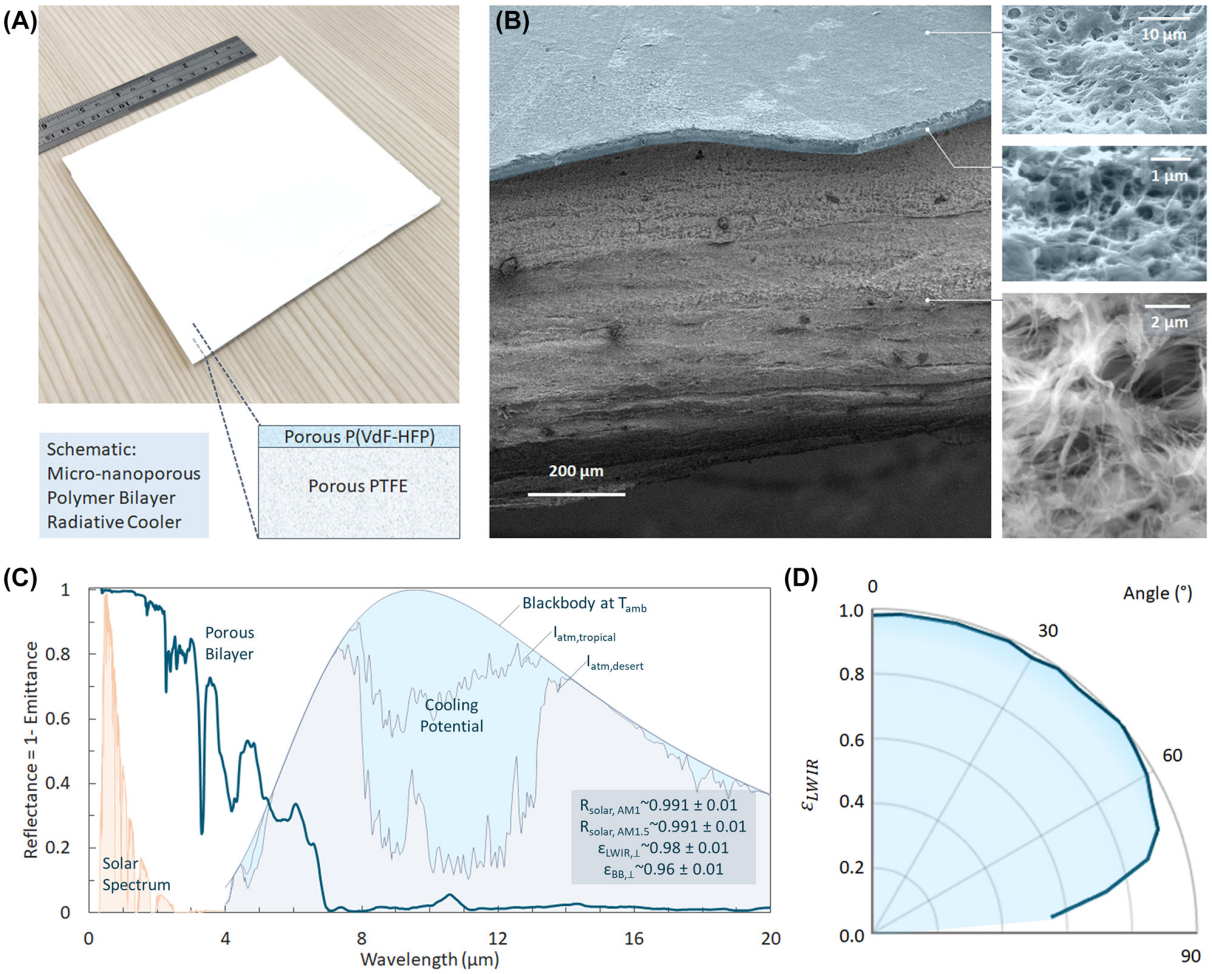
Microstructural and optical characterization of ePTFE-P(VdF-HFP) bilayer. (A) Photograph of a 15  cm × 15 cm ePTFE-P(VdF-HFP) bilayer radiative cooler. (B) (Left) Scanning electron micrograph of the bilayer with both the top surface and cross-section in view. (Right) Magnified view of the porous P(VdF-HFP) top surface and cross section, and the PTFE cross section. P(VdF-HFP) layer is colored blue. (C) Reflectance spectrum of ePTFE-P(VdF-HFP) radiative cooler in the solar and TIR wavelengths. The background shows a normalized solar spectrum, and atmospheric irradiance showing cooling potentials for different sky conditions. (D) Angular *ɛ*_LWIR_ profile of the radiative cooler from 0° to 85°.

To fabricate the bilayer porous polymer, we considered different fabrication pathways. Although PTFE’s optical properties make it highly appealing, its chemical inertness, resistance to solvents, and incompatibility with melt processing make it particularly difficult to process. One pathway to make porous PTFE is to sinter PTFE particles under heat and pressure [26]. While that can yield high reflectances [27], [28], sintering often leads to fusion of particles and porosities of ∼40 % [29], leaving too few air voids for efficient scattering. Consequently, sintered PTFEs reported in the literature need to be several mm thick to achieve near-unity reflectance [24], [27], [30]. An alternative is to use PTFE in its expanded form (ePTFE). When heated to high temperatures and rapidly stretched, PTFE expands into a highly porous (∼65 %), nanofibrillar form, with fibril sizes comparable to those in our simulations (Figures 1E and 2) [31], [32]. This indicates the potential for a high solar reflectance. Indeed, ePTFE is known for its brilliant white color, and is commercially available, at very large scales [32].

Given its promise, we explored ePTFE as the solar reflective underlayer for our design. 0.8–1 mm thick ePTFE sheets were purchased from EqualSeal, and examined under a Quanta 200 FEG Scanning Electron Microscope. As shown in Figure 2B, the ePTFE comprises of numerous 0.2–0.5 μm thick fibrils branching from nodes. As expected from the optical properties and scattering efficiencies (Figure 1C and E), the microstructure imparts an exceptional *R*_solar_ of 0.992 for ∼1 mm thick films (Figure S4B). However, as expected, *ɛ*_LWIR,⊥_(*λ*) is low, and is calculated to be 0.86 (Figures S4 and 6).

To augment *ɛ*_LWIR_, we then proceeded to create a porous P(VdF-HFP) layer on the ePTFE using a highly scalable phase inversion method [1]. A precursor solution of acetone, P(VdF-HFP) and water in 8:1:1 mass ratio was coated on the ePTFE using an applicator. The rapid evaporation of acetone, followed by phase separation of P(VdF-HFP) and water, and eventual evaporation of water, left nano porous P(VdF-HFP) films with microscale surface roughness on the ePTFE (Figure 2B). As expected from our theoretical results (Figure 1F), the surface roughness and the effective medium behavior due to the nanopores lead to a considerably higher emittance than a solid P(VdF-HFP) film (Figure S3B). To find the best combination of *R*_solar_ and *ɛ*_LWIR_, we coated different film thicknesses of the precursor solution on the ePTFE, and took spectral reflectance measurements. Figure S6A shows that for a *nominal film* thickness (corresponding to applicator setting rather than physical value) of ∼75–100 μm, the porous film reaches a plateau at *ɛ*_LWIR_ ∼ 0.98, compared to ePTFE’s 0.86, for only a 0.001 drop in *R*_solar_. Higher thicknesses reduce *R*_solar_ with little gain in *ɛ*_LWIR_, so we aimed for a nominal film thickness in the ∼75–100 μm range for the bilayer design.

The superwhite bilayer porous polymer resulting from our design process is shown in Figure 2A. As shown in Figure 2B, the porous P(VdF-HFP) topcoat has 5 μm surface features at the top, and ∼0.2–0.8 μm pores within, while the underlayer comprises of 0.2–0.5 μm PTFE nanofibrils. Notably, the 75–100 μm nominal film thickness corresponds to a ∼30 μm thin P(VdF-HFP) topcoat above the 0.8–1 mm thick ePTFE. The structural and material properties of the two components described earlier complement each other to yield an exceptional optical performance (Figure 2C). The bilayer porous polymer has a near-ideal *R*_solar_ of 0.991 at near normal incidence, and likely greater at high angles due to a higher effective thickness for incident light. The near-normal LWIR emittance *ɛ*_LWIR,⊥_ is calculated at 0.981. Angular radiometric measurements (Figure 2D, SI, Section 2) revealed the emittance stays nearly constant up to 60° angle from the surface normal, and only drops below 0.90 after 75°, which is desirable as the atmospheric irradiance is high near the horizon [33], [34]. The hemispherical emittance *ɛ*_LWIR_ is calculated at 0.96. Collectively, the measured values of *R*_solar_ and *ɛ*_LWIR_ represents a near-ideal combination, and validates our design approach towards ideal optical performance (Figure 1). We note here that the *R*_solar_ could be potentially increased to 0.996, or higher, by using ePTFE layers that are 2 mm or thicker (SI, Section 5). Here we keep our optimizations limited to a 1 mm layer, as that is better in terms of cost and through-plane thermal conductance to the cooling target.

## 5 Outdoor performance tests

**Figure 3:**
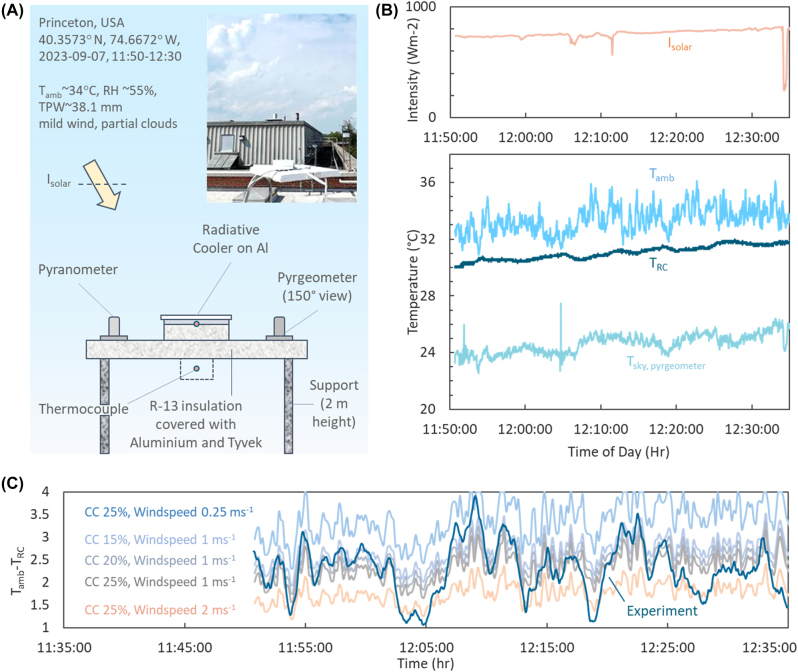
Experimental validation of radiative cooling performance. (A) Schematic of the experimental setup, with an inset showing a photograph of the physical setup in the test location. (B) (Top) Solar irradiance measured by the pyranometer. (Bottom) Temperatures of the ambient air *T*_amb_ and radiative cooler *T*_RC_, and the effective graybody sky temperature *T*_sky, pyrgeometer_ derived from pyrgeometer readings, as a function of time. (C) Comparison of experimentally overserved sub-ambient cooling *T*_amb_ − *T*_RC_ and theoretical values calculated based on various cloud covers (CC) and windspeeds.

We tested the steady state radiative cooling performance of our bilayer design under an open sky on a late summer day Princeton, USA. The experimental setup is shown in Figure 3A and detailed in the SI, Section 3. Figure 3B shows the results for the first ∼45 min of the experiment. The data for the remaining duration, which saw clouds intermittently block direct sunlight, is shown in the SI, Section 3. As shown, the radiative cooler achieves a sub-ambient temperature throughout the period, with an average sub-ambient cooling of 2.31 °C observed for the first 45 min, and 2.26 °C observed over the entire experiment.

While the observed sub-ambient cooling is lower than those of previously reported radiative coolers [1], [10], this is attributable to meteorological conditions (Figure 3A). During the first part of the experiment (Figure 3B), a moderately high solar intensity of ∼750 Wm^−2^, a high total precipitable water (TPW) ∼38.1 mm [35], a partial cloud cover of ∼20–25 %, and fluctuating windspeeds of 0.5–2 ms^−1^, made sub-ambient radiative cooling quite difficult [36], [37], [38]. Indeed, in such scenarios, sub-ambient cooling cannot be achieved unless solar reflectances are sufficiently high [39], [40]. The mean sky temperature calculated from pyrgeometric measurements was ∼25 °C, only 8 °C cooler than the mean *T*_amb_. This indicated a radiative cooling potential of 50 Wm^−2^ [7] or less, since the pyrgeometer does not register sky radiation at grazing incidences. Calculations performed with MODTRAN^®^ using *T*_amb_ and TPW as inputs yielded cooling potential of 50–60 Wm^–2^ for cloud covers of 25–15 % [1], [38].

We compared the observed sub-ambient cooling *T*_amb_ − *T*_RC_ with theoretical calculations involving solar absorption by the radiative cooler, MODTRAN^®^ sky irradiances based on TPW, *T*_amb_ and cloud cover, and different windspeeds (SI, Section 3). Importantly, using MODTRAN^®^ enabled spectral calculations using the optical properties in Figure 2C and D. The results, presented in Figure 3C, show that our experimental results lie within the bounds of the different cloud cover and windspeed scenarios. Indeed, much of the variation in *T*_amb_ − *T*_RC_ appear to be due to windspeed, with differences between average observed and theoretical *T*_amb_ − *T*_RC_ being within 0.17–0.4 °C for windspeeds of 1–2 ms^−1^ and cloud covers of 20–25 %. The fact that we observe radiative cooling in far-from-ideal conditions and cooling potentials is due to the high *R*_solar_ and *ɛ*_LWIR_ of the bilayer radiative cooler. Furthermore, the design’s exceptionally high *R*_solar_ is corroborated by the remainder of the experiment (Figure S1 **)**, which shows that even large fluctuations in solar intensity (∼500–700 Wm^−2^) due to shading by clouds has no effect on the sub-ambient cooling. In other words, sunlight does not discernibly impact cooling performance.

## 6 Potential applications and outlook

The optical performance of the porous P(VdF-HFP)-ePTFE bilayer made is complemented by its scalability. ePTFE can be made at large scales through mechanical extrusion, followed by uniaxial or biaxial stretching at high temperatures [41]. The P(VdF-HFP) toplayer can likewise be made by a scalable phase inversion technique used to make polymer filtration membranes. These processes are both established, and suitable for industrial production (Figure S8). The fact that the bilayer design uses commercially available polymers also adds to its potential for scalable manufacturing and use.

The high optical performance and scalability of the porous P(VdF-HFP)-ePTFE bilayer makes it attractive for use in a range of applications, such as cooling HVAC systems [42], passive ventilation [43], direct cooling of vehicles [44], infrastructure [45], water harvesting from air [46], and freezing desalination [47], and potentially, cooling rooftops of buildings [1]. A preliminary cost analysis (SI, Section 6) shows that the areal cost of the design is ∼ 20 US$/m^2^. This is reasonable for device-based or high-end applications listed above. For building envelopes, however, the high cost (∼2×) relative to those of commercially available cool roof paints represents a potential barrier towards adoption. Nonetheless, we note that the potential cooling energy savings in buildings, which can be ∼2× that of cool roof paints [48], and the potentially long service life given the fluoropolymer composition of our design [49], make the bilayer cost-effective and promising for use in the built environment when cost-returns in the long run are considered.

We end by noting that although the porous P(VdF-HFP)-ePTFE bilayer attains a near-ideal optical parameters for radiative cooling, it may be possible to augment it further. A major issue that remains to be addressed is material usage – the low solar refractive index of PTFE (*n*–1.38) and the potentially unoptimized morphology of ePTFE – mean that a thickness of ∼1 mm is needed to achieve high *R*_solar_. This leads to more material usage, which in turn might impact costs and potential adoption.

One way to minimize thickness could be to replace the lower part of the ePTFE underlayer with a thinner, porous polymer, where the polymer has a higher refractive index for more efficient scattering. Potential materials include cellulose acetate (*n*–1.47), which can be easily phase inverted [1], [11], [50] and mylar (*n*–1.6). With regard to morphology optimization, we note that ePTFE fabrication parameters can be tuned to change both the nanofibrous structure and porosity [41], which in turn would impact backscattering of sunlight. One particularly intriguing possibility is to tailor the morphology to maximize scattering at *λ*∼2 μm, where ePTFE shows some absorption (Figure S4). It may also be possible to morphologically alter the porous P(VdF-HFP) top coat to further enhance *ɛ*_LWIR_, particularly at high angles (Figure 2D). Along with the exploration of potential applications, these possibilities open avenues for future fundamental and applied research.

## Supplementary Material

Supplementary Material Details
