# Supplementary material for: Porous polymer bilayer with near-ideal solar reflectance and longwave infrared emittance
Source: Nanophotonics. 2024 Jan 17;13(5):669–77. doi: 10.1515/nanoph-2023-0707 (PMC11501546; doi:10.1515/nanoph-2023-0707)
Supplement: Supplementary file 1 — Supplementary Material Details [file j_nanoph-2023-0707_suppl_001.docx]

Supporting Information for Porous Polymer Bilayer with Near-ideal Solar Reflectance and Longwave Infrared Emittance

Anson Tsang, ^1^ Nithin Jo Varghese, ^†1^ Mathis DeGeorges, ^†1,2^ Jyotirmoy Mandal^1,3^*

^1^Department of Civil & Environmental Engineering, Princeton University, New Jersey, USA

^2^Institut National des Sciences Appliquées de Lyon, Lyon, France

^3^Princeton Materials Institute, Princeton University, New Jersey, USA

# Section 1: Definition of Solar Reflectance, LWIR Emittance and Cooling Potential

The solar reflectance $R_{solar}$ of a material is defined as:

$R_{solar} =\frac{\int_{0}^{\infty} I_{solar}\left( \lambda\right)\cdot R\left( \lambda\right)d\lambda}{\int_{0}^{\infty} I_{solar}\left( \lambda\right)d\lambda}$ (S1)

where λ is the wavelength, I_solar_ (λ) is the AM1 or ASTM G173 Global solar intensity spectrum and $R(\lambda)$ is the material’s spectral reflectance.

The directional LWIR emittance $\varepsilon_{LWIR}(\theta)$ is defined as:

$\varepsilon_{LWIR}(\theta) =\frac{\int_{8 \mu m}^{13 \mu m} I_{BB}\left( T, \lambda\right)\cdot\epsilon\left( \theta,\lambda\right)d\lambda}{\int_{8 \mu m}^{13 \mu m} I_{BB}\left( T, \lambda\right)d\lambda}$ (S2)

where the 8 and13 μm bounds represent the LWIR atmospheric transmittance window, $I_{BB}\left( T, \lambda\right)$ is the spectral intensity emitted by a blackbody at temperature T (assumed to be 25˚C) and $\varepsilon\left( \theta,\lambda\right)$ is the material’s spectral directional thermal emittance.

The near-normal emittance $\varepsilon_{LWIR,\perp}$ corresponds to the case where $\theta$ is 8°, as it is difficult to practically make accurate measurements at near-normal incidence.

The hemispherical emittance $\varepsilon_{LWIR}$ is given by:

$\varepsilon_{LWIR}=\frac{\int_{0}^{\frac{\pi}{2}} \varepsilon_{LWIR}\left( \theta\right)\cdot I_{BB}\cos\theta\sin\theta d\theta}{\int_{0}^{\frac{\pi}{2}} I_{BB}\cos\theta\sin\theta d\theta}$ (S3)

The cooling potential is defined as:

$I_{BB}\left( T_{amb} \right)-I_{sky}(T_{amb},TPW)$ (S4)

where $T_{amb}$ is ambient temperature, TPW is total precipitable water, and $I_{sky}$ is the downwelling atmospheric irradiance.

# Section 2: Optical Measurements

Spectral reflectance, absorptance, transmittance measurements were taken using a Bruker Invenio-X Fourier-transform spectrophotometer. For specular reflectance measurements, a Harrick Seagull variable angle accessory set at 10° angle of incidence was used. For diffuse transmittance or reflectance measurements, integrating spheres were used. Solar wavelengths were measured with a custom built PTFE integrating sphere equipped with silicone and germanium photodiodes, and Spectralon SRS-99 reflectance standards. Thermal infrared (TIR) wavelengths were measured with a custom-built gold integrating sphere equipped with a mercury-cadmium-telluride detector and Infragold reflectance standards. For the integrating spheres, the angle of incidence of light was about 10°. Notably, the port sizes of the spheres were kept small to reduce errors in measurement.

For the optical measurements shown in **Fig. 1B-D**, ~125 μm thick films of each polymer in bulk, smooth form was used. The respective specularity of each material is listed in **Table S1**. The spectra were then weighted by the AM1.5 NIR-to-SWIR solar spectrum.

For the directional emittance measurements in **Fig. 2D**, a 1”x1” bilayer radiative cooler sample was placed vertically and held at 100°C using a constant temperature hot plate. A FLIR T685 thermal camera was used to image the heated sample at 5° intervals from 0°-85° angles of emittance. Since the bilayer sample was expected to have a diffuse reflectance, the reflected ambient radiative temperature for each angle was measured using a diffuse gold sample. Emittance at each angle was calculated using the equation:

$\varepsilon_{LWIR,\theta}=\frac{T_{camera}^{4}-T_{ambient}^{4}}{T_{surface}^{4}-T_{ambient}^{4}}$ (S5)

The total hemispherical emittance was calculated from $\varepsilon_{LWIR,\theta}$ using a discretized version of Eq. S3.

$\varepsilon_{LWIR}=\frac{\int_{0}^{\frac{\pi}{2}} \varepsilon_{LWIR,\theta}\sin\left( \theta\right)\cos\left( \theta\right)d\theta}{\int_{0}^{\frac{\pi}{2}} 1\sin\left( \theta\right)\cos\left( \theta\right)d\theta}=\frac{\sum_{i} \frac{\epsilon_{i}+\epsilon_{i+1}}{2}\sin\left( \theta_{i} \right)\cos\left( \theta_{i} \right)\Delta\theta_{i}}{\sum_{i} \sin\left( \theta_{i} \right)\cos\left( \theta_{i} \right)\Delta\theta_{i}}$ (S6)

**Table S1:** Bulk polymer materials tested for its transmittance and reflectance. The surface finish of the materials dictated it’s specularity and hence its corresponding method of measurement.

| **Material** | **Surface** |
| --- | --- |
| PTFE | Smooth |
| ECTFE | Matte |
| Silicone | Smooth |
| P(VdF-HFP) | Matte |
| PVC | Smooth |
| Mylar | Smooth |
| Cellulose Acetate | Smooth |
| Epoxy | Matte |
| Polyethene | Matte |
| Acetal | Matte |
| PMMA | Smooth |

# Section 3: Outdoor Field Tests

In the past experiment illustrated in **Fig. 3A**, a 15 cm x 15 cm ePTFE-P(VDF-HFP) film, serving as a radiative cooler, was adhered to a 1 mm thick 6061-aluminum sheet and mounted on R13 insulation foam board purchased from Home Depot. This board was covered with Tyvek and aluminum foil featuring 93% solar reflectance to minimize solar heating or radiative cooling of the setup. The entire arrangement was placed approximately 2 meters above the light-colored roof to avert local hotspots. The location was the Princeton University Engineering Quadrangle in Princeton, New Jersey, USA, and the time was 2023-09-07, 11:50-15:00 local time.

Two k-type thermocouples were employed, one for measuring $T_{RC}$ and another shaded and below the setup to measure $T_{amb}$. Importantly, our measured $T_{amb}$ matched readings from the local weather station (35). An Apogee SP510 pyranometer and an Apogee SL510 pyrgeometer were installed on the installation to record solar and atmospheric TIR irradiances respectively. Conductive losses were deemed negligible due to the high thermal resistance of the foam board. All sensors were linked to an Omega Octpro datalogger that collected data throughout the experiment, after allowing the setup to reach steady state temperature.

Steady state temperature measurements and solar/thermal irradiance were documented for roughly three hours, with **Fig. 3B** depicting the initial approximately 45 minutes. The subsequent period, experiencing intermittent cloud cover obstructing direct sunlight, is shown below. There appears to be no relationship between the variations in $I_{solar}$ and $\Delta T=T_{amb}-T_{RC}$, indicating that the exceptionally high $R_{solar}$ of the radiative cooler makes solar heating negligible.


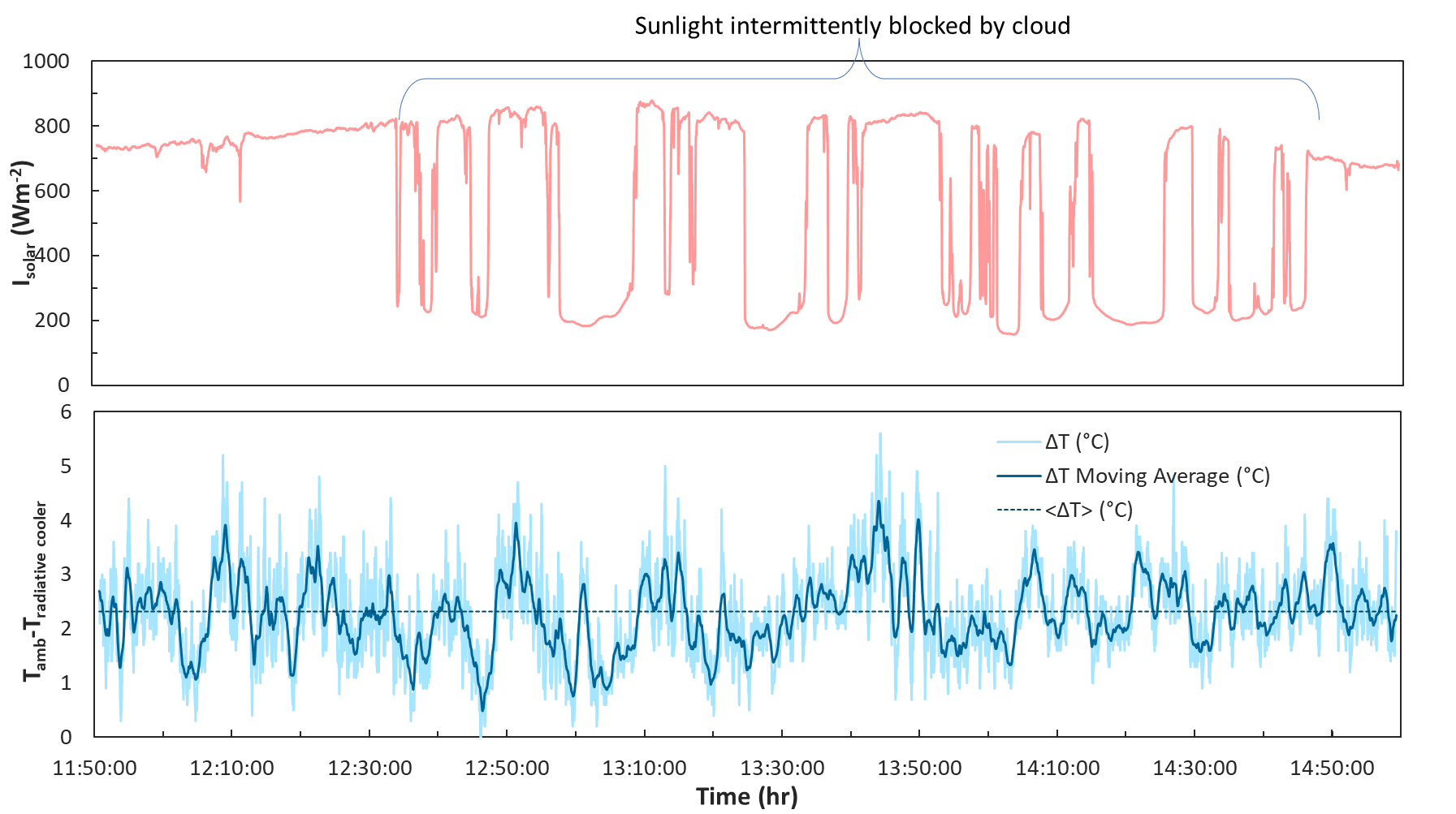


***Figure S1****.* $I_{solar}$ *and* $\Delta T=T_{amb}-T_{RC}$ *for the entire experiment, including the period when direct sunlight incident on the sample was intermittently blocked by clouds.*

Theoretical Analysis

To check if our experimental results are expected, an energy balance analysis was done. A convective heat transfer coefficient *h* of 5-15 Wm^-2^K^-1^ is used, which corresponds to 0.25-2 ms^-1^ of windspeed. The list conditions are listed below:

***Table S2****: List of different environmental conditions considered for steady state temperature balance.*

| Case | Wind Speed [m/s] | Cloud Coverage [%] |
| --- | --- | --- |
| 1 | 0.25 | 25 |
| 2 | 1 | 15 |
| 3 | 1 | 20 |
| 4 | 1 | 25 |
| 5 | 2 | 25 |

Conduction was ignored due to the high thermal resistance of the insulation foam. The temporal data collected from the datalogger is binned and averaged for every 10 s. The spectral emissivity information was used from the sample measurements.

$Q_{conv}+\int Q_{rad}\left( \lambda\right)d\lambda=0$ (S7)

$Q_{rad}\left( \lambda\right)=\varepsilon_{RC}\left( \lambda\right) \left[ I_{bb}\left( T_{RC},\lambda\right)-I_{sky,net}\left( T_{amb},\lambda\right) \right]-\left( 1-R_{sol} \right)I_{sol}$ (S8)

Due to the presence of cloud coverage, separate thermal irradiances had to be considered for both the clear sky and cloud covered sky. MODTRAN® was used to calculate the clear sky irradiance $I_{sky}$ and cloudy sky irradiance $I_{cloud}$, using a cumulus cloud model. The TPW was extracted from a publicly available archive [1]. The cloud coverage *F* was manually estimated. The net sky radiation was calculated as follows.

$I_{sky,net}=\left( 1-F_{cloud} \right)I_{sky}+F_{cloud}I_{cloud}$ (S9)

The radiative potential of a blackbody is calculated using the Planck’s equation for thermal radiation.

$I_{bb}= \frac{2hc^{2}}{\lambda^{5}}\frac{1}{e^{\frac{hc}{\lambda k_{B}T}}-1}$ (S10)

The theoretical value for $T_{amb}-T_{radiative cooler}$ is calculated at every datapoint to satisfy the conservation equation and is shown in **Fig. 3C** against the measured value.

# Section 4: Effective Medium Behavior of Porous P(VdF-HFP) – Theory and Experiments

The phase inversion technique for fabricating porous P(VDF-HFP) yields pore sizes where effective medium approximations such as Maxwell-Garnett approximations, are valid.

The Maxwell Garnett Effective Medium Approximation is defined by the dielectric permittivity of its constituents ( $\varepsilon_{air}$,$\varepsilon_{PVDF}$)as well as the volume fraction of the inclusion (air in this case):

$\varepsilon_{eff}^{MG}=\varepsilon_{P(VdF-HFP)}\frac{\left( \varepsilon_{air}+2\varepsilon_{P(VdF-HFP)} \right)+2f\left( \varepsilon_{air}-\varepsilon_{P(VdF-HFP)} \right)}{\left( \varepsilon_{air}+2\varepsilon_{P(VdF-HFP)} \right)-f\left( \varepsilon_{air}-\varepsilon_{P(VdF-HFP)} \right)}$ (S12)

Subsequently the complex refractive index can be calculated from the following relationship:

$\varepsilon_{eff}^{MG}=\left( n_{eff}+i\kappa_{eff} \right)^{2}$ (S13)

which can be input into the Fresnel equation to give us the surface reflectance at normal incidence of the effective medium:

$R=\frac{\left( n_{eff,}-1 \right)^{2}+{\kappa_{eff}}^{2}}{\left( n_{eff}+1 \right)^{2}+{\kappa_{eff}}^{2}}$ (S11)

As shown in **Fig. S2**, by introducing air as a secondary phase in the porous medium, the effective refractive index is reduced below that of the bulk polymer, which should theoretically lead to a higher emissivity as shown in **Fig. 1F**.


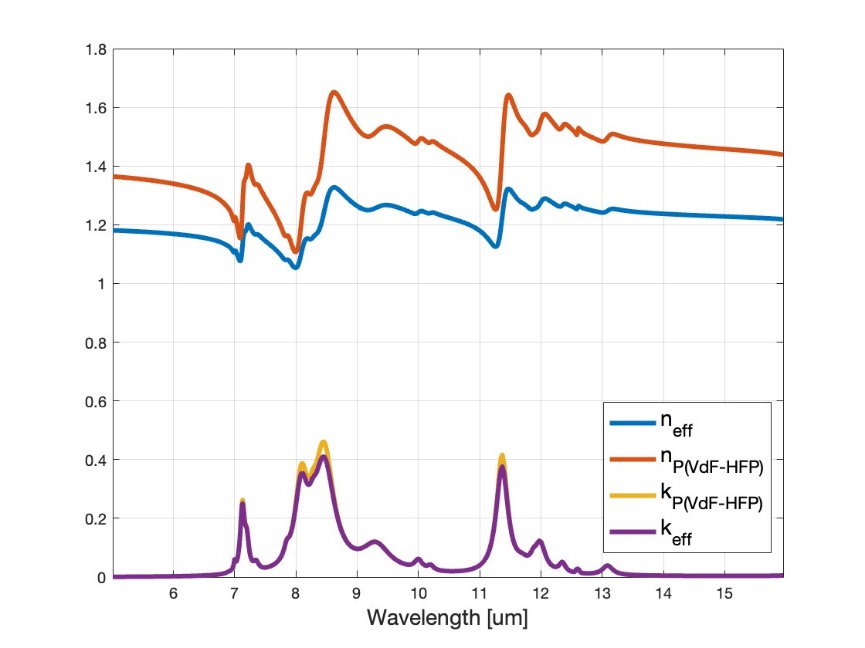


***Figure S2.*** *The real complex and complex refractive indexes of solid and porous P(VdF-HFP) calculated using Maxwell Garnett effective medium theory is shown, highlighting the effective reduction in refractive index with a porous air medium.*

The theoretical calculations are well substantiated by experiments. To test if the effective medium behavior holds, we coated a ~25 µm thin film of solid P(VdF-HFP) on ePTFE, and compared its reflectance with our bilayer design. As clear from **Fig. S2**, the porous P(VdF-HDP) achieved a significantly higher $\varepsilon_{LWIR,\perp}$across the entire thermal IR range when compared to its solid form when tested using the integrating sphere setup with FTIR. Thus, the porous microstructure is able to enhance the emissivity of P(VdF-HFP) by rendering the material’s refractive index closer to that of air.


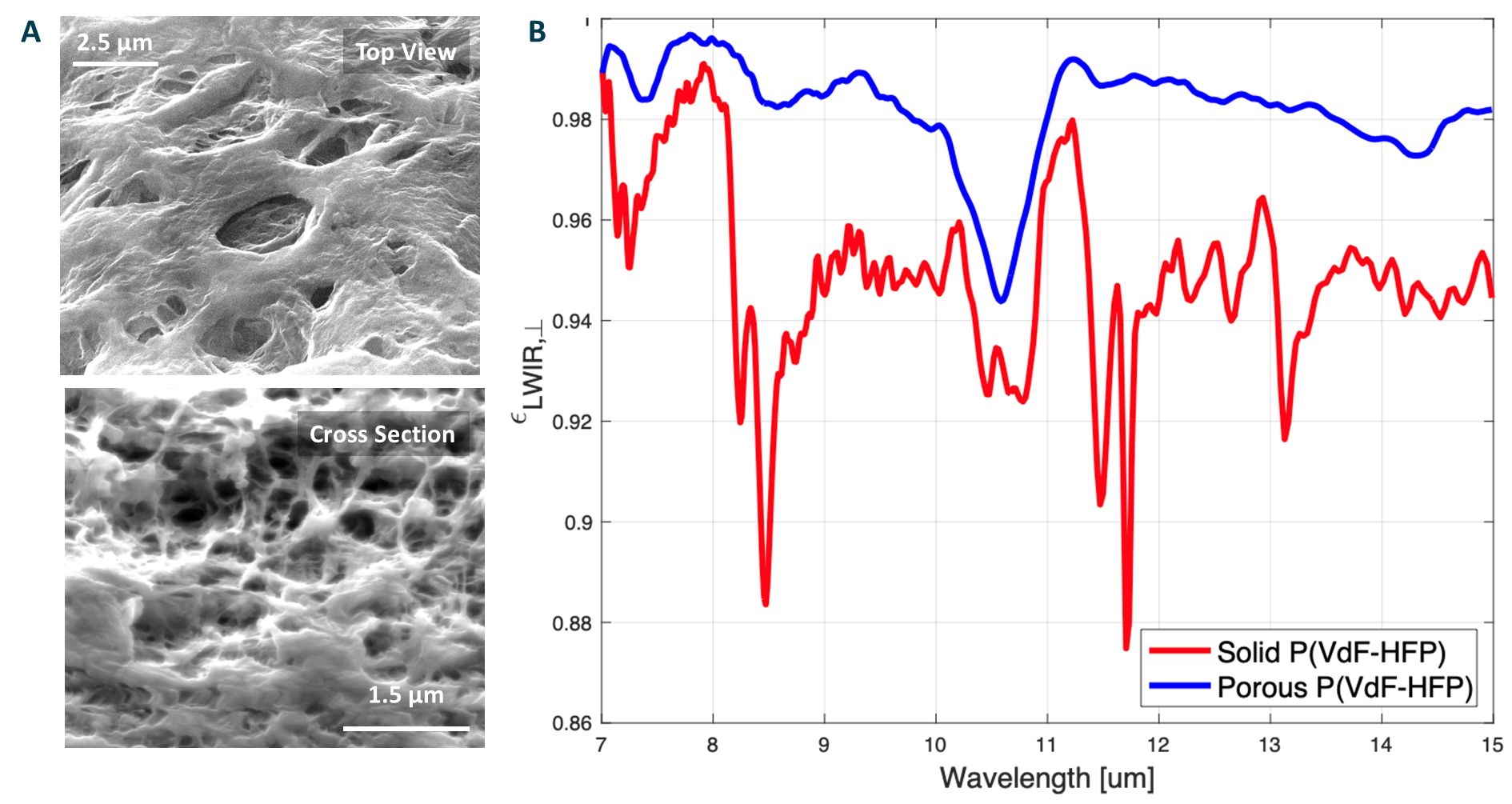


***Figure S3****.* ***(A)*** *SEM images of the porous P(VdF–HFP) rough surface and nanoporous cross section.* ***(B)*** *Near-normal LWIR emittance of solid and porous P(VdF-HFP) topcoats on ePTFE.*

# Section 5: Effect of P(VdF-HFP) Topcoat and ePTFE Underlayer Thickness on $\boldsymbol{R}_{\boldsymbol{solar}}$ and $\boldsymbol{\varepsilon}_{\boldsymbol{LWIR}}$

**Fig. S4** shows the nanofibrous structure and spectral reflectance of ePTFE. As evident, the scattering by the nanofibers, coupled with the very low intrinsic absorption, lead to an outstanding $R_{solar}$ of 0.992. However, PTFE’s lack of molecular vibrational modes in the LWIR means that even minimal scattering by the nanofibers leads to a low $\varepsilon_{LWIR}$ of ~0.86, necessitating our bilayer design.


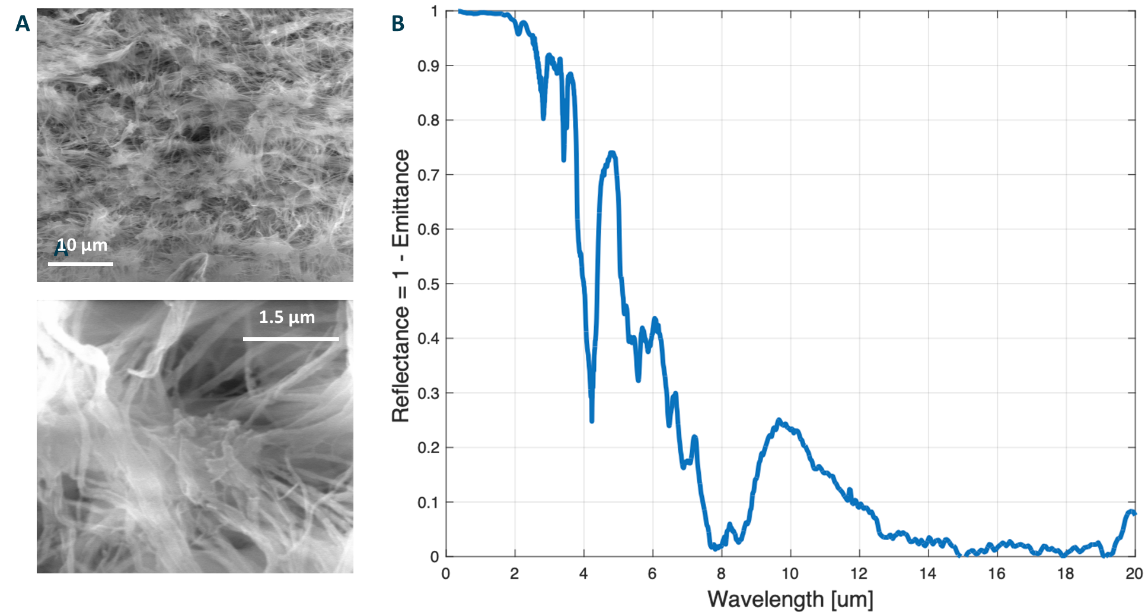


***Figure S4****.* ***(A)*** *Scanning electron micrographs of the cross section of ePTFE taken at different magnifications. The fibrous microstructure allows for efficient scattering.* ***(B)*** *Spectral reflectance of ~1 mm thick ePTFE.*

The optical properties of the porous P(VdF-HFP) top layer were also analyzed. The transmittance of a thin film is shown in **Fig. S5**. The solar reflectance is relatively low for the 93 μm sample measured, for the thickness used in the bilayer design (30 μm), $R_{solar}$ would be even lower. This is acceptable, however, as the primary function of porous P(VdF-HFP) is to enhance $\epsilon_{LWIR}$.

***Figure S5.*** *Transmittance of 93 μm porous P(VdF-HFP) film from 0.4-15.4 μm.*

To choose the optimal thickness of the P(VdF-HFP) layer, different thicknesses were coated on bare ePTFE. As **Fig. S6B** shows, when the nominal thickness of the P(VdF-HFP) precursor layer increases to 100µm, $\varepsilon_{LWIR}$ rises sharply, but afterwards, starts to plateau. Similarly, $R_{solar}$ is fairly constant up to 100µm, but then drops afterwards. The optimal thickness that achieves both high $\varepsilon_{LWIR}$ *and* $R_{solar}$ is between 70-100µm, and we chose it as the nominal thickness target for our bilayer design.


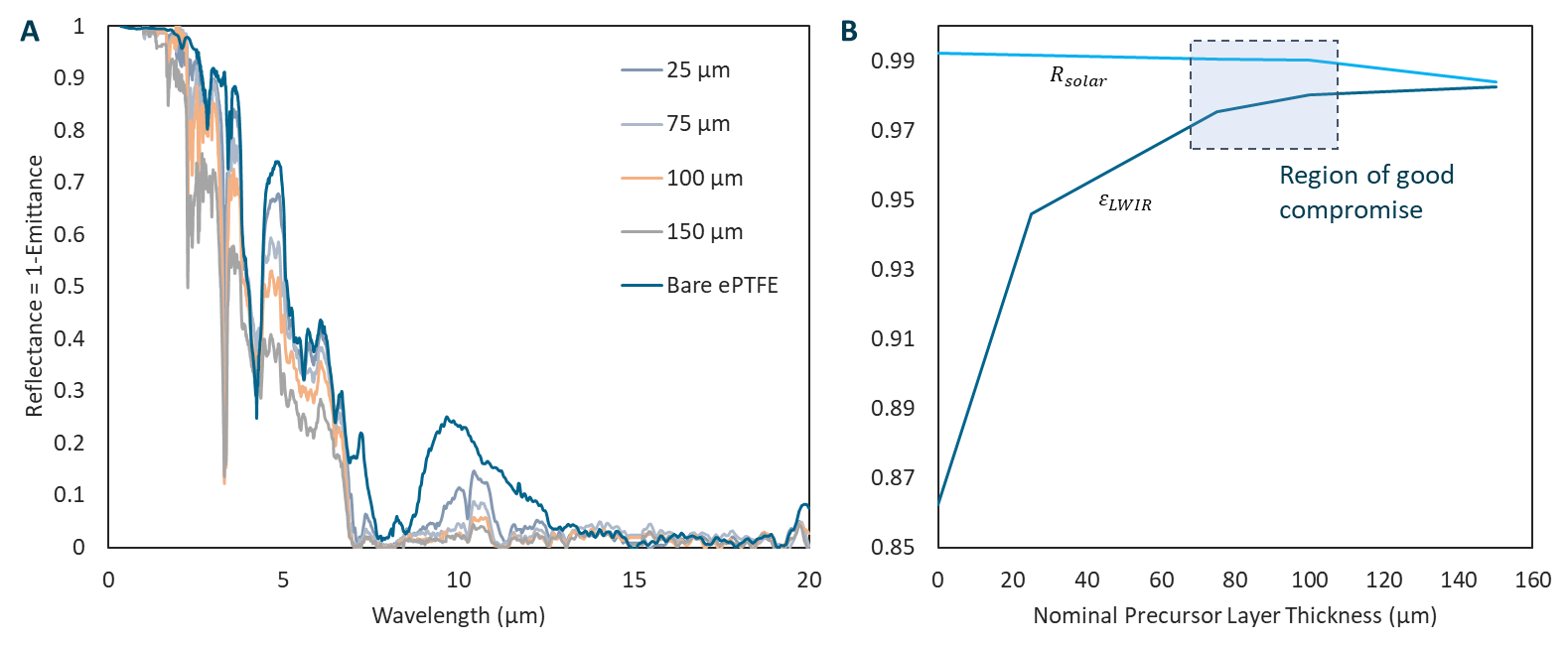


***Figure S6. (A)*** *Reflectance spectrum of the ePTFE coated different thicknesses of P(VdF-HFP) from 0.3-20 μm.* ***(B)*** *The* $R_{solar}$ *and* $\varepsilon_{LWIR}$ *of ePTFE coated different thicknesses of P(VdF-HFP).*

Although the optimal performance our coating attains is an $R_{solar}\sim0.991$, a near-normal $\varepsilon_{LWIR,\perp}\sim0.98$, and hemispherical $\varepsilon_{LWIR}\sim0.96$, $R_{solar}$ can be further increased by increasing the thickness of the ePTFE layer. Measuring changes in reflectance in the order of 0.001 with integrating spheres is difficult, so instead, we measured the transmittance of a ~1mm ePTFE film, and ~2 mm ePTFE films, with a grating-based UV-VIS spectrophotometer (STS Series by Ocean Optics). Signal integration over long times allowed us to capture very small changes in ultraviolet-to-visible transmittance of a strong beam from a 250W tungsten halogen lamp. Since PTFE has no intrinsic absorption in the UV-to-visible solar wavelengths, changes in transmittance would correspond to changes in reflectance. This, in turn, allows us to estimate the reflectances of thick films of eTPFE. **Fig. S7** shows that $R_{solar}\approx0.997$ for ~2 mm thickness of ePTFE, compared to 0.992 for ~1mm thickness. Accounting for absorption by porous PVDF top layer, which reduces $R_{solar}$ to 0.991 over a 1 mm thick ePTFE, we anticipate that a similar porous PVDF film on 2 mm thick ePTFE would achieve a solar reflectance of 0.996. Using thicker layers of ePTFE could improve the performance further, but it is quite difficult to estimate the improvement with confidence. In any case, such optimizations are rather academic, as such thick ePTFE layers are too costly to be useful.

**Figure S7.** *Reflectance of varying thicknesses of ePTFE from 0.35-2.5μm.*

# Section 6: Cost Analysis

In this section, we estimate the material and fabrication costs of the bilayer radiative cooler. Using commercially available pricing data [2]–[4], we estimate the materials cost of our bilayer structure to be ~11 USD/m^2^ (ePTFE substrate: 10 USD/m^2^, P(VdF-HFP) powder: 0.28 USD/m^2^, Acetone: 0.43 USD/m^2^). Note that because the ePTFE is already produced commercially at scale, the price reflects the actual spot cost of procuring the material. Fabricating the P(VdF-HFP) topcoat on the ePTFE film can be done with a roll-to-roll film coating method with the phase inversion process integrated into it. Given the simplicity and mechanical nature of the process [5], we expect the manufacturing costs (without considering capital costs) to be on the order of the material cost. As such, we expect the total cost to be around or less than USD 20/m^2^.

By comparison, cool roof paints price ranges from 4-12 USD/m^2^, with highly reflective paints falling on the high end of this estimate. However, their optical properties ($\varepsilon_{LWIR}$~90%, $R_{solar}$~70-86%) are markedly lower than the bilayer radiative cooler. This can lead to our design having a cooling energy savings of as much as 2x relative to white paints [6]. Furthermore, the hydrophobicity and UV stability of both P(VdF-HFP) and PTFE [7], [8] gives its excellent weathering properties. P(VdF-HFP) and PTFE are already commonly used in coatings for large scale civil structures such as bridges and water-tanks, with service lives that can be over a decade as opposed to ~5 years for cool roof paints. Therefore, ePTFE-P(VdF-HFP) bilayer has the potential to achieve higher energy savings over a longer service life, which would make it more cost effective than white paints in the longer run.

Beyond buildings, the radiative cooler is particularly promising for high-end applications, such as fluidic cooling of HVAC systems[9], direct cooling of vehicles [10], infrastructure [11], water harvesting from air [12], [13], and freezing desalination [14]. For such applications, the price per unit area requirements is less stringent than for buildings, and the bilayer radiative cooler could foreseeably be used in such applications.

# Section 7: Porous P(VdF-HFP)-ePTFE Bilayer Fabrication

**Fig. S8** shows the bilayer fabrication process. The nanofibrous ePTFE is made using an established thermo-mechanical process that involves extrusion of the PTFE resin, calendaring, and the uniaxial or biaxial stretching of the film under heat. The porous P(VdF-HFP) film is made using a highly scalable phase inversion technique used to make polymer filtration membranes. Both of these processes are suitable for industrial production at large scales.


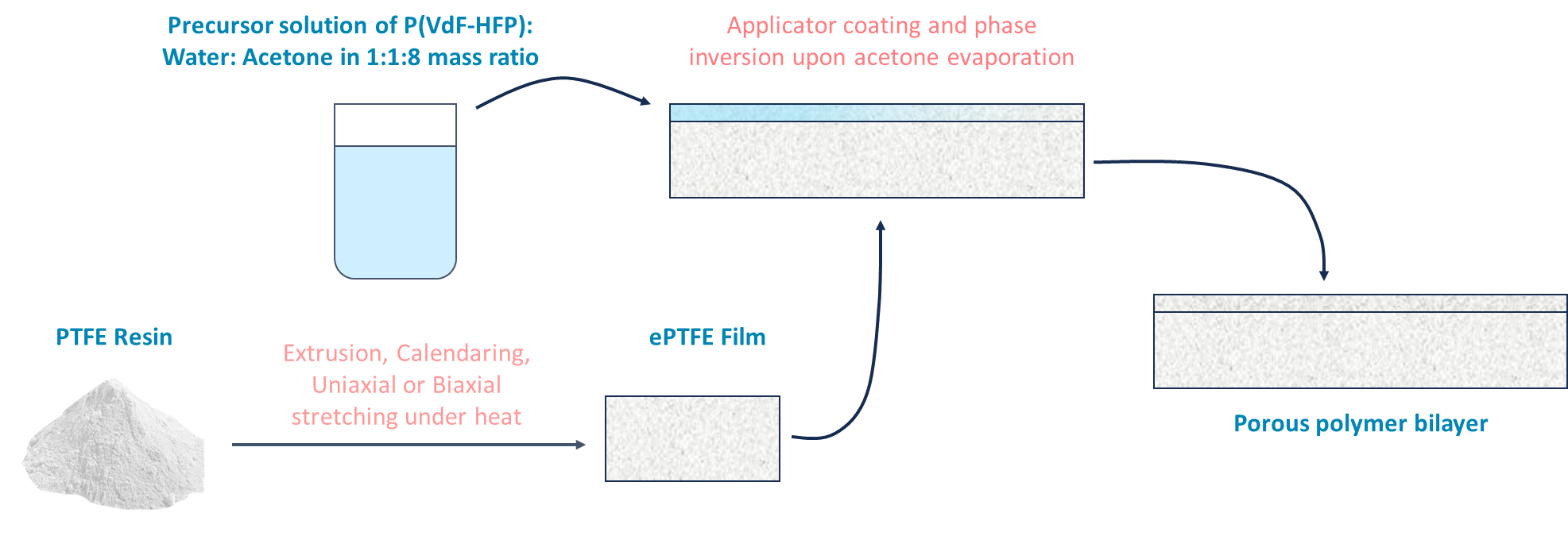
***Figure S8.*** *Fabrication process of the Porous P(VdF-HFP)-ePTFE Bilayer.*

# Section 8: FDTD Simulations

To investigate the effect the porous structure on the optical properties of the bilayer, Finite-Difference Time-Domain (FDTD) simulations were performed using Lumerical. In the solar wavelengths, scattering efficiencies of 3D circular voids and fibers of different diameters using a total-field scattered-field source. The results are shown in Fig. 1E.

In the LWIR, spectral reflectance $R(\lambda)$ for light incident at normal incidence from the air above was simulated for semi-infinite solid P(VdF-HFP) slabs with triangular surface patterns. The surface pattern is shown in Fig. S7. To reduce computational loads, a 2D simulation was performed in line with past works [5], [15]. Periodic boundary conditions in the X direction, perfectly matched layer boundaries in the Y direction, a planar wave source emitting LWIR wavelengths downwards, and a frequency domain power monitor above the plane wave source was used. Figs. 1F shows the results.


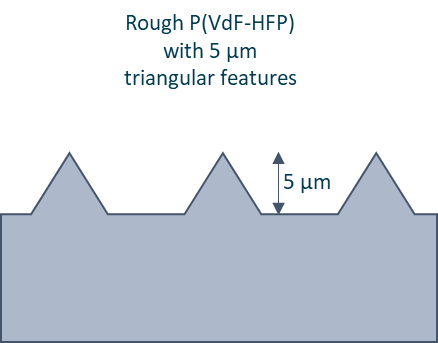


***Figure S8.*** *2D Domain Setup for Lumerical FDTD simulation highlighting the 5 μm triangular features.*

# References

[1] Anthony Wimmers and Chris Valden, ‘MIMIC-TPW Version 2’, MIMIC-TPW Version 2. Accessed: Oct. 30, 2019. [Online]. Available: http://tropic.ssec.wisc.edu/real-time/mtpw2/product.php

[2] Alibaba, ‘Unm Factory Wholesale Price Waterproof Breathable Eptfe High Efficiency Filter Membrane Ptfe Clean Room Filtration Film - Buy Eptfe,Ptfe Film,Ptfe Membrane Product on Alibaba.com’. Accessed: Oct. 16, 2023. [Online]. Available: https://www.alibaba.com/product-detail/UNM-Factory-Wholesale-Price-Waterproof-Breathable_1600139941766.html?spm=a2700.galleryofferlist.p_offer.d_title.7f837a1aDlbjiU&s=p

[3] Alibaba, ‘High Quality Pvdf Powder Polyvinylidene Fluoride For Lithium Battery Pvdf Coating Powder’. Accessed: Oct. 16, 2023. [Online]. Available: https://www.alibaba.com/product-detail/High-Quality-Pvdf-Powder-Polyvinylidene-Fluoride_1600934583736.html?spm=a2700.galleryofferlist.topad_classic.d_title.6bc62ae4WiSVRQ

[4] Mike, ‘Acetone price index’, businessanalytiq. Accessed: Oct. 16, 2023. [Online]. Available: https://businessanalytiq.com/procurementanalytics/index/acetone-price-index/

[5] J. Mandal *et al.*, ‘Hierarchically porous polymer coatings for highly efficient passive daytime radiative cooling’, *Science*, vol. 362, no. 6412, p. 315, Oct. 2018, doi: 10.1126/science.aat9513.

[6] A. Baniassadi, D. J. Sailor, and G. A. Ban-Weiss, ‘Potential energy and climate benefits of super-cool materials as a rooftop strategy’, *Urban Climate*, vol. 29, p. 100495, Sep. 2019, doi: 10.1016/j.uclim.2019.100495.

[7] Arkema, ‘KYNAR® & KYNAR FLEX® PVDF Performance Characteristics and Data’, Arkema. Accessed: Jul. 23, 2019. [Online]. Available: https://www.extremematerials-arkema.com/en/product-families/kynar-pvdf-family/download-performance-characteristics-data-brochure

[8] Labsphere, ‘Space Grade Spectralon® Diffuse Reflectance Material’, Labsphere. Accessed: Oct. 16, 2023. [Online]. Available: https://www.labsphere.com/product/space-grade-spectralon-diffuse-reflectance-material/

[9] R. Fortin, J. Mandal, A. P. Raman, and S. Craig, ‘Passive radiative cooling to sub-ambient temperatures inside naturally ventilated buildings’, *Cell Reports Physical Science*, vol. 4, no. 9, p. 101570, Sep. 2023, doi: 10.1016/j.xcrp.2023.101570.

[10] R. Levinson, ‘Using solar availability factors to adjust cool-wall energy savings for shading and reflection by neighboring buildings’, *Solar Energy*, vol. 180, pp. 717–734, Mar. 2019, doi: 10.1016/j.solener.2019.01.023.

[11] Z. Cui, C. Guo, and D. Zhao, ‘Energy-saving and economic analysis of passive radiative sky cooling for telecommunication base station in China’, *Build. Simul.*, vol. 15, no. 10, pp. 1775–1787, Oct. 2022, doi: 10.1007/s12273-022-0894-z.

[12] M. Zhou *et al.*, ‘Vapor condensation with daytime radiative cooling’, *Proceedings of the National Academy of Sciences*, vol. 118, no. 14, p. e2019292118, Apr. 2021, doi: 10.1073/pnas.2019292118.

[13] T. M. J. Nilsson and G. A. Niklasson, ‘Radiative cooling during the day: simulations and experiments on pigmented polyethylene cover foils’, *Solar Energy Materials and Solar Cells*, vol. 37, no. 1, pp. 93–118, Apr. 1995, doi: 10.1016/0927-0248(94)00200-2.

[14] X. Huang, J. Mandal, J. Xu, and A. P. Raman, ‘Passive freezing desalination driven by radiative cooling’, *Joule*, vol. 6, no. 12, pp. 2762–2775, Dec. 2022, doi: 10.1016/j.joule.2022.10.009.

[15] M. Chen *et al.*, ‘Designing Mesoporous Photonic Structures for High-Performance Passive Daytime Radiative Cooling’, *Nano Lett.*, Feb. 2021, doi: 10.1021/acs.nanolett.0c04241.
